# Heat Shock Proteins in Benign Prostatic Hyperplasia and Prostate Cancer

**DOI:** 10.3390/ijms23020897

**Published:** 2022-01-14

**Authors:** Weronika Ratajczak, Michał Lubkowski, Anna Lubkowska

**Affiliations:** 1 Department of Functional Diagnostics and Physical Medicine, Pomeranian Medical University, Żołnierska 54, 71-210 Szczecin, Poland; weronika.ratajczak@pum.edu.pl; 2 Student Research at the Chair and Department of Functional Diagnostics and Physical Medicine, Pomeranian Medical University, Żołnierska 54, 71-210 Szczecin, Poland; mi.lubkowski@gmail.com

**Keywords:** heat shock proteins, prostate cancer, benign prostatic hyperplasia, prostate diseases

## Abstract

Two out of three diseases of the prostate gland affect aging men worldwide. Benign prostatic hyperplasia (BPH) is a noncancerous enlargement affecting millions of men. Prostate cancer (PCa) in turn is the second leading cause of cancer death. The factors influencing the occurrence of BPH and PCa are different; however, in the course of these two diseases, the overexpression of heat shock proteins is observed. Heat shock proteins (HSPs), chaperone proteins, are known to be one of the main proteins playing a role in maintaining cell homeostasis. HSPs take part in the process of the proper folding of newly formed proteins, and participate in the renaturation of damaged proteins. In addition, they are involved in the transport of specific proteins to the appropriate cell organelles and directing damaged proteins to proteasomes or lysosomes. Their function is to protect the proteins against degradation factors that are produced during cellular stress. HSPs are also involved in modulating the immune response and the process of apoptosis. One well-known factor affecting HSPs is the androgen receptor (AR)—a main player involved in the development of BPH and the progression of prostate cancer. HSPs play a cytoprotective role and determine the survival of cancer cells. These chaperones are often upregulated in malignancies and play an indispensable role in tumor progression. Therefore, HSPs are considered as one of the therapeutic targets in anti-cancer therapies. In this review article, we discuss the role of different HSPs in prostate diseases, and their potential as therapeutic targets.

## 1. Introduction

The cells of organisms are constantly exposed to external and internal factors that damage them. In response to environmental and metabolic factors and the occurrence of pathophysiological stress conditions, including high temperature, hypoxia, infectious agents (bacterial and viral), UV light, toxic substances as well as inflammatory mediators, the expression level of heat shock proteins (HSPs) increases [1]. The main function of HSPs is to control the process of folding the correct structure of proteins; thanks to their chaperoning role they are able to bind and interact with many cellular factors. Due to the molecular masses, these proteins found in mammals have been classified and assigned to six main families: HspH (Hsp110, 100 kDa or higher); HspC (Hsp90, 83–90 kDa); HspA (Hsp70, 70 kDa); DNAJ (Hsp40, 40 kDa); HspB (small HSPs, sHSPs, 10–30 kDa) and the chaperonin families: HspD/E (Hsp60/Hsp10) and CCT (cytosolic chaperonin TCP1 ring complex, TRiC) [2,3,4,5]. HSPs are ubiquitously present in a variety of cellular compartments. Their functions differ depending on the type of HSP and the physiological state during which they appear in the cell. The action of chaperones is ATP dependent, with the exception of HSPs belonging to the small HSP family. Under normal physiological conditions, eukaryotic cells have basic levels of HSPs called constitutive HSPs and at these levels they act as “housekeeping” proteins. However, upon exposure to stress factors, the expression level significantly increases, contributing to the generation of a physiological response to the factors in question, which is called the “heat shock response” (HSR). The factors regulating HSR are heat shock factors (HSF), 1, 2, 3, 4 and HSFY (located on the human Y chromosome), which together with HSPs form a complex and keep them inactive [6]. The role of the major HSP regulator has been attributed to HSF-1, which controls the expression of HSP genes. Upon the detection of stress, HSF-1 becomes activated, detaches from the HSP and, by binding to the specific regions of DNA, the sequences of heat shock elements (HSEs) in the promoter region of HSP genes, activates protein transcription. The result of this process is an increase in the level of free HSPs, which in turn deactivates HSF-1 in response to a feedback response [7]. Importantly, increased HSF-1 expression is also observed in various types of cancer, where it regulates the mechanisms of various types of cell death [8]. Moreover, the activity of HSF-1 is also related to tumor progression and influences their metastatic potential [9,10]. HSPs interact in different ways with molecules involved in the pathways of programmed survival or death, and this occurs at specific stages. It has been postulated that the overexpression of HSPs prevents apoptosis induced by various factors [11,12], and their endogenous levels are sufficient to control this process. Moreover, it is believed that the inhibition of the expression of most HSP members is sufficient to “sensitize” cells to apoptosis [13,14,15].

Prostate cancer is the second most common neoplastic disease, and was the fifth most common cause of cancer deaths among men worldwide in 2020 [16]. The pharmacological treatment of PCa, also in the presence of metastases, mainly consists of lowering the concentration of androgens, which is effective in most cases. However, to achieve better survival rates in patients, chemotherapy (docetaxel) is used. Androgen-deprivation therapy in patients with high-risk non-metastatic prostate cancer lasts three years and is additionally combined with radiotherapy. The latest data from the meta-analysis of the third phase of clinical trials prove that the effective therapy is the supplementation of ADT with abiraterone and prednisolone, together with enzalutamide, which should be considered a new standard treatment for high-risk non-metastatic prostate cancer. It has been shown that combination therapy is associated with significantly higher rates of metastasis-free survival, compared with ADT alone [17]. Moreover, one of the new therapeutic perspectives is the use of the PPAR inhibitor, olaparib, which acts synergistically with the HSP90 inhibitor (AT13387), which has so far been confirmed in clinical trials in mCRPC patients [18]. The greatest problem in the treatment of prostate cancer is the resistance of these cells to treatment aimed at inducing apoptosis, controlling signaling pathways responsible for cell proliferation or modulating the activity of the androgen receptor. In the treatment of patients, personalized methods of treatment using the latest technologies, “omics” technologies that regulate the mechanisms of the functioning of neoplastic cells at the molecular and epigenetic level, seem to be helpful [19]. Prostate cancer cells are constantly exposed to proteotoxic stress. This state forces the cell to activate the cytoprotective mechanisms, in which are involved, among others, heat shock proteins, which are now also a therapeutic target, including CRPC cancer [20].

The histological changes in the prostate gland, an increase in the number of cells, and thus an increase in its tissue mass, progresses with the aging process. The gland enlarges and its structure hardens, which puts pressure on the urethra and the appearance of the clinical symptoms of the disease. Benign prostatic hyperplasia (BPH) is one of the most common etiological factors of lower urinary tract symptoms (LUTS) in men. The prevalence of histological BPH increases with age and in men aged 81–90 years it is even 90%. In turn, the prevalence of LUTS in men aged 40–59 increases from 40% to 70% in men over 80 years of age. LUTS/BPH causes a significant deterioration of health and quality of life in men and is the most common disease in this population [21]. Along with the increase in the incidence of BPH, the treatment options have increased, but they are still not fully satisfactory [22,23].

## 2. HSPs and Cancer Cells

In normal cells under physiological conditions, in a state of undisturbed homeostasis, cytoprotective mechanisms operate, thanks to which they are able to survive the stressful conditions. Cells that are not exposed to stress factors show enough HSP expression to protect their proteome and ensure cellular homeostasis (proteostasis). A number of significant changes take place in neoplastic cells, including, at the level of activity of the transcription factors and metabolic activity, glycolysis levels, lipid metabolism or amino acid metabolism [24,25].

Cancer cells are exposed to high levels of proteotoxic stress. They enter stress response pathways for survival and proliferation and become dependent on stress-induced HSPs. Moreover, the intracellular homeostasis of neoplastic cells is regulated by the increased expression of HSPs. In this case, the HSP-mediated cytoprotection of cancer cells takes place by inhibiting apoptosis, which is important for the proliferation, invasiveness and metastasis of tumor cells [4]. In addition, the high level of HSP expression promotes the folding of oncoproteins, which ensures their stability and reduces the likelihood of their proteolytic degradation.

The expression of HSPs is induced in response to a variety of physiological and environmental factors, including anti-cancer chemotherapy. Such a strategy allows the cells to survive even under lethal conditions. Importantly, in neoplastic diseases, HSP expression is usually increased, which has been confirmed in gastric cancer [26], breast cancer [27], endometrial cancer, ovarian cancer [28,29], gastrointestinal cancers [30], lung cancer [31] and in prostate cancer [32].

Many signaling pathways play an important role in the pathogenesis of neoplastic diseases, and their incorrect regulation leads to changes in the cell phenotype and disturbances of such important processes, such as the regulation of the cell cycle, growth, death, differentiation and cell adhesion [33]. In eukaryotic cells, two complementary processes aimed at the degradation of native intracellular proteins can be distinguished: lysosomal degradation, including macroautophagy, and proteasomal degradation. Lysosomes mainly break down extracellular proteins that enter the cell through endocytosis, or, in the case of macroautophagy, also the intracellular proteins under strong cellular stress. Proteasomes, in turn, are responsible for the controlled degradation of proteins with lower molecular weights, including signaling proteins with a short half-life and misfolded proteins [34]. Current therapeutic strategies for neoplastic diseases mainly aim to induce apoptosis in these cells by genotoxic action or the inhibition of their proliferation. Proteasome inhibitors lead to an increase in the transcription of genes encoding proteins from the HSP90, HSP70, HSP40, HSP28, HSP APG-1 and mitochondrial HSP75 families. These proteins play a significant role in the development of mechanisms of resistance to therapeutic compounds. Cancer cells treated with proteasome inhibitors aim to compensate for the decreased activity of this protease by increasing its synthesis and the synthesis of chaperone molecules [35].

## 3. The Androgen Receptor in the Development of Prostate Cancer and Benign Prostatic Hyperplasia

The androgen receptor (AR) is expressed in tissues, such as the prostate, skeletal muscles, liver and the central nervous system (CNS). Under physiological conditions, in the prenatal period, the androgen receptor, through the action of androgens, is responsible for the sexual differentiation of the fetus and changes in adolescence. On the other hand, in adult men, androgens, in addition to regulating the function of a normal (healthy) prostate, also affect the maintenance of libido, spermatogenesis, muscle mass and strength, bone mineral density and erythropoiesis [36]. Male sex hormones act through an axis involving the synthesis of testosterone (T) in Leydig cells in the testes and in small amounts in the adrenal glands, its transport to target tissues, and then intracellular conversion by 5α-reductase (and its two isoenzymes) [37] to a more active metabolite, 5α-dihydrotestosterone (DHT). The synthesis of androgens is regulated by the action of the luteinizing hormone (LH) produced by the pituitary gland. In turn, LH secretion is stimulated by the hypothalamic gonadotropin-releasing hormone (GnRH) [38]. The biological effect of androgens is mediated by their binding to AR, which induces its transcriptional activity [39].

The androgen receptor belongs to the nuclear receptors and is a specific ligand-dependent transcription factor. Its structure is similar to other steroid receptors: the estrogen receptor (ER), progesterone receptor (PR), glucocorticoid receptor (GR), mineralocorticoid receptor (MR) and thyroid hormone receptors (TR). Moreover, similar to other steroid receptors, the ligand-free AR is localized in the cytoplasm and forms a complex with heat shock proteins by interacting with the ligand binding domain [40]. The androgen receptor is an 11 kDa protein. The gene encoding the AR is located on the X chromosome (Xq11-12) and consists of 8 exons. In the structure of AR, 4 regions can be distinguished: the N-terminal domain (NTD) (NH2 terminal transactivation domain) encoded by exon 1, the DNA-binding domain (DBD) (exon 2 and 3), the hinge region and the C-terminal domain LBD (ligand-binding domain) (exons 4–8) [41]. The NTD region contains between 19–25 glutamine repeats (CAG repeats), which vary between males, resulting in amino acid variability in the AR. The length of the polymorphic CAG repeat sequence affects the transcriptional activity of AR. It has been confirmed that men with shorter glutamine repeats, although within the normal range, are more likely to develop prostate cancer and develop symptomatic benign prostatic hyperplasia [42]. In addition, an AF-1 activating domain, needed for maximal AR activity, is distinguished in the NTD region. In turn, in the LBD region, an AF-2 activating domain was distinguished, which forms the coregulator binding site. In addition, AF-2 also mediates the interaction between the N-terminal domain and the C-terminal domain (N/C interactions) [43,44]. Moreover, the LBD region is the testosterone and dihydrotestosterone binding site. The consequence of LBD binding to the ligand is a change in the AR conformation and its translocation to the cell nucleus, in which a dimer is formed that connects to ARE (androgen response element) in the promoter region of the genes critical for the growth and development of a healthy prostate, as well as prostate cancer cells, but also important factors for the terminal differentiation of PSA (prostate specific antigen), or human kallikrein 2 (hK2) [41]. This mechanism inhibits the proliferation of prostate cells, despite the high level of growth factors secreted by the stromal cells [45]. The proper development of the prostate gland depends on AR activity. Mesenchymal cells (fibroblasts and myocytes) expressing AR are then stimulated by androgens and secrete growth factors, e.g., the insulin-like growth factor (IGF), fibroblast growth factor (FGF), epidermal growth factor (EGF), which, by paracrine signaling, stimulate the neighboring cells to grow and develop the entire gland [46,47]. Under physiological conditions, the androgen-stimulated stromal cells of the prostate secrete growth factors, thanks to which the homoeostasis of the epithelial part of the gland is maintained, which prevents prostate regression and the initiation of the neoplastic process. The androgen receptor also plays a key role in the development of prostate cancer [41] and is also believed to be involved in the excessive proliferation of prostate cells observed during the development of BPH [48]. During the neoplastic process, a change occurs, the abnormal epithelial cells become independent of the regulation by stromal cells, but are still autonomously stimulated by AR. Then, AR does not act as a suppressor of cell proliferation, but plays the role of an oncogenic growth stimulator [49].

The androgen receptor is expressed exclusively in the nucleus of prostate cells, which has been confirmed both in healthy prostate tissue and in tissue with benign hyperplasia. The presence of AR has been observed in luminal cells, in fibromuscular stromal cells and in the epithelial cells of blood vessels. However, the expression of AR in basal cells in the glandular epithelium of the prostate has not been confirmed [50]. On the other hand, in prostate tissue with diagnosed prostate adenocarcinoma, a significant increase in cells showing AR immunoexpression was confirmed in neoplastic cells, non-neoplastic glandular epithelial cells and in the peritumoral zone and in the interglandular part of the stromal cells [51]. The dependence of the development of prostate cancer (PCa) on the effects of androgens and AR was first proved by Hyggins and Hodges [52]. This discovery influenced the subsequent development of ablative hormone therapy for prostate cancer supporting the inhibition of neoplastic tumor growth [53,54].

Most prostate tumors are androgen-sensitive tumors and their growth depends on the transcriptional activity of the androgen receptor. The androgen receptor and factors modulating its activity are of great importance in the development of prostate cancer. About 90% of diagnosed prostate cancers are androgen-dependent, and thus one of the most effective therapies is hormone therapy that reduces serum androgen levels and androgen receptor inhibition. However, due to mutations and the continuous expression of AR during tumor progression, hormone therapy often fails [39,55]. In the study on a murine model, the loss of AR from the cells of the fibromuscular stroma leads to the inhibition of the development of the intraepithelial neoplastic process PIN (prostatic intraepithelial neoplasia), the reduction of epithelial proliferation and the remodeling of the extracellular matrix (ECM). The infiltration of pro-inflammatory cells and the process of creating new vessels are also reduced [56].

An example is castration-resistant prostate cancer (CRPC), which has shown the reactivation of AR receptor signaling. This situation results from the overexpression of AR, which has been confirmed in patients with prostate cancer [57] who develop tumor progression at the time of using ADT (androgen deprivation therapy) [58].

**Figure 1:**
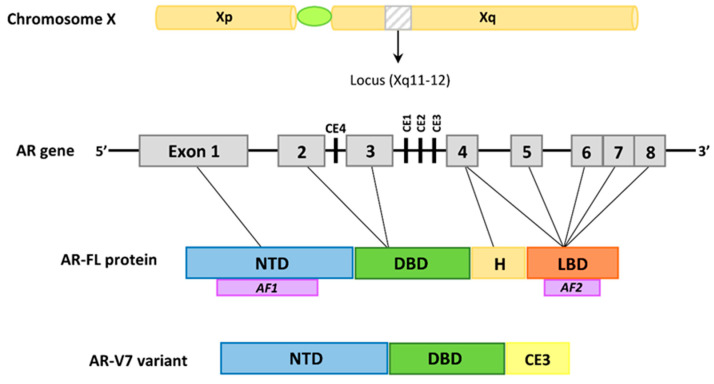
The structure of the AR-FL and AR-V7 variants. The AR gene is located on the X chromosome (Xq11-12) and it is composed of 8 exons. Exon 1 encodes the amino-terminal domain (NTD), which contains the AF1 activating domain. Exons 2 and 3 encode the DNA-binding domain (DBD). The 5′ region of exon 4 forms the hinge (H) region, which contains the nuclear localization signal, whereas the 3′ region of exon 4 and exons 5–8 encode the ligand-binding domain (LBD), which contain the AF2 transactivation region. AR-V7, also known as AR3, is truncated at the end of exon 3. It lacks the LBD, and contains 16 unique amino acids from cryptic exon 3 (CE3). AR-V7 is constitutively active.

One of the mechanisms involved in the variability of AR receptor signaling and ADT failure is the presence of AR splicing variants. To date, more than 20 different AR variants have been identified, the common feature of which is the loss of fragments of the C-terminal domain, resulting in a shortening or loss of LBD [41], which is a therapeutic target for enzulamide. Therefore, the AR variants lacking this domain are active despite the lack of androgenic activity. The most common variant, and thus the most important one, is the splicing variant AR-V7 (androgen receptor splice variant 7, also called AR3), which, in the cells of patients with hormone-refractory PCa (HRPC), shows a 20-fold higher expression, than in the cells of patients not previously treated with hormone-naive PCa [59]. AR-V7 is truncated at the end of LBD and contains 16 amino acids from the cryptic exon 3b (CE3). The resulting protein is constitutively active in the absence of androgens. Additionally, it has also been confirmed that AR-V7 affects the growth of PCa cells in cell lines (Figure 1) [60].

In the immunohistochemical study conducted by Sharp et al. [61], it was confirmed that the expression of the AR-V7 protein in the material collected from patients with primary PCa is very rare (<1%), while in the patients undergoing androgen therapy (ADT), the expression of AR-V7 was confirmed in 75% of the cases. In turn, in patients with CRPC cancer, nuclear protein expression was found in 94% of the cases, which additionally correlated with the expression of AR-FL (full-length androgen receptor). Moreover, the AR-V7 protein is heterogeneously expressed, and this difference is mainly seen in secondary tumors/metastases in the same patient. These data indicate an association of AR-V7 expression with the occurrence of different (drug) resistance mechanisms in one patient. This also suggests the need for alternative therapeutic modifications to reverse the hormonal resistance of AR-V7 in patients with PCa [61].

It is also believed that the AR-V7 variant can play the role of a biomarker of prostate cell resistance to available hormonal treatment, including that with the use of second-generation androgen receptor signaling inhibitors (ARSi), such as abiraterone acetate (Abi) and enzalutamide (Enza) [62]. It is also very important that patients with metastatic castration-resistant prostate cancer (mCRPC), who have the AR-V7 variant and receive Enza or Abi, have a worse progression-free survival (PFS) and overall survival (OS) than patients without AR-V7 expression. It is also found that the presence of the AR-V7 variant is not associated with a significant resistance to taxanes [63]. Importantly, it is also easily determined, among others, in the material from liquid biopsies, e.g., from circulating tumor cells, and in whole blood RNA. This variant is of particular clinical importance due to the high level of expression in individuals with advanced stage prostate cancer. The androgen receptor is a signaling protein (substrate, “client” protein) for various HSPs. The AR not bound to a specific ligand is found in the cytoplasm of target cells for steroid hormones, where it can interact with chaperone proteins (including HSP70 and HSP90) and co-chaperone proteins [64,65].

## 4. The Characteristics of HSP90

HSP90s, molecular chaperones, in cells play an essential role in the process of protein folding, and in addition are responsible for the process of the conformational maturation and assembly of a diverse group of substrates, client proteins, including kinases, hormone receptors, transcription factors and membrane proteins. The protein isoforms of the HSP90 family are distributed in various places in the cell, including in the cytoplasm (HSP90α, HSP90β), the endoplasmic reticulum (ER) (GRP94) and the mitochondria (TRAP1) [66].

**Figure 2:**
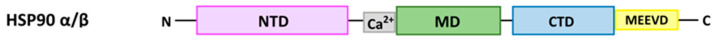
Domain structure of the HSP90 protein. The N-terminal domain (NTD) mediates ATP binding, the middle domain (MD) is involved in the hydrolysis of ATP and the binding of HSP90 to substrates (client protein) and the C-terminal domain (corboxy-terminal domain, CTD) is responsible for HSP90 dimerization, containing the motif Met-Glu-Glu-Val-Asp (MEEVD).

HSP90s have an ATPase domain. In addition, there are three highly conserved domains in the structure of the HSP90 monomer: the N-terminal domain (amino-terminal domain, NTD) mediating ATP binding [67], the middle domain (MD), which is involved in ATP hydrolysis and binding HSP90 with substrates (client protein) [68] and the C-terminal domain (corboxy-terminal domain, CTD) responsible for HSP90 dimerization, containing the sequences (motif) Met-Glu-Glu-Val-Asp (MEEVD) or KDEL, important for interaction with co-chaperone proteins, which contain tetratricopeptide repeat (TPR) domains [69]. The presence of amino acid sequences depends on the HSP90 isoform and their cellular localization MEEVD is found in HSP90 α and β (present in the cytosol), and KDEL in GRP94 (glycoprotein present in the ER) (Figure 2) [70].

The N-terminal domain of the HSP90 contains an ATP-binding pocket, which is highly similar to the evolutionarily conserved family of protein domains, GHKL (Gyrase subunit B, Hsp90, Histidine Kinase, MutL) [71]. The ATP-binding site is necessary in carrying out the ATPase activity-dependent attachment of client proteins to HSP90. The ATPase function and activity of the NTD domain is modulated by the MD domain, which binds NTD-specific ATP γ-phosphate. In addition, it is a binding site for client proteins and co-chaperones.

In eukaryotes, the structure of HSP90s includes a region of variable length and amino acid composition that connects the NTD and MD domains, the charged linker region (CR) [72]. The CR region is of particular importance for the structural flexibility of these two domains; it participates in the generation of a docked state in which the NTD domain is stable towards the MD domain, but also mediates the generation of the undocked domain locations in which the NTD domain can change its position. Additionally, the CR region affects the activation of client proteins, cell viability and their stress tolerance [72].

The C-terminal domain is necessary in the HSP90 dimerization process. In this process, two C-terminal helices form a four-helix bundle [73]. In addition to the region involved in homodimerization, there is also a calmodulin-binding site (calmodulin-binding domain), which can bind various types of proteins in a Ca^2+^-dependent way. Additionally, this domain can modulate the structure and functions of HSP90 [74]. 

In addition, the CTD domain has also been shown to be the second ATP-binding site, except that this site is only accessible after having occupied the ATP-binding site in the NTD domain. In addition, this site binds purine and pyrimidine nucleotides; the C-terminal specific nucleotides are: UTP and GTP, which affects the autophosphorylation of HSP90. The nucleotides that bind to the CTD-binding site, which do not require the previous occupation of the N-terminal site, are TNP nucleotides and pyrophosphate [75].

**Figure 3:**
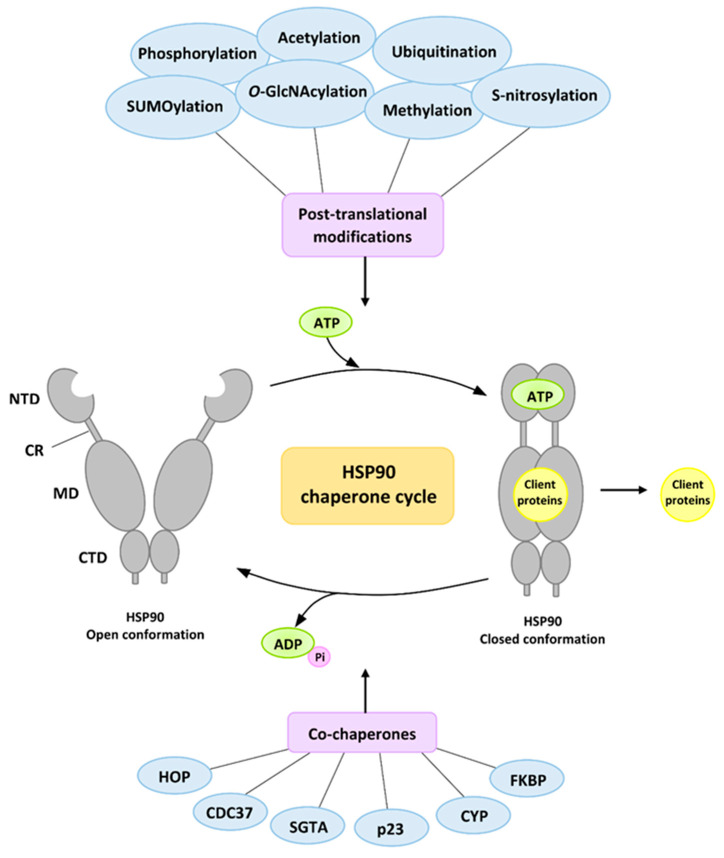
The HSP90 chaperone cycle. At the beginning of the cycle, HSP90 has an open conformation, and only the C domain dimerizes. ATP binding and an ordered series of conformational changes allow it to adopt a closed conformation, which is N-terminally dimerized. After ATP hydrolysis, HSP90 returns to the open conformation and is ready to begin another ATPase cycle. During the chaperone cycle, the client proteins are activated. This cycle is tightly regulated by various post-translational modifications and the action of the co-chaperones proteins.

The regulation of the activity and function of HSP90 is precisely controlled and takes place at various levels, and includes transcriptional regulation, post-translational modification and the action of co-chaperones proteins (Figure 3) [76]. 

At the transcriptional level, the HSP90 expression is induced by the action of HSF-1, which is also its client protein. It is now shown that along with HSP70, HSP90 binds to HSF-1 and keeps it inactive. When chaperones are needed in the cell to perform their functions, they are disconnected from HSF-1, which enables the induction of the transcription of HSP-coding genes and their increase in expression [77]. In the context of prostate cancer, a strong weakening of HSF-1 expression reduces the proliferation of prostate cancer cells. The use of an inhibitor that directly targets HSF-1 (Direct Targeted HSF1 Inhibitor (DTHIB)) affects the degradation of nuclear HSF-1. Moreover, it is then possible to suppress the signaling pathways related to the AR and its splicing variant (AR-V7). DTHIB can also act independently of AR and reduce the progression of PCa in murine models, including the highly aggressive NEPC (neuroendocrine prostate cancer) [78].

The post-translational modifications (PTMs) modulating HSP90 functions include, among others, phosphorylation, SUMOylation, acetylation, methylation, O-GlcNAcylation, ubiquitination and S-nitrosylation [79]. The phosphorylation of HSP90 takes place mainly on serine (Ser) residues, but also on threonine (Thr) and tyrosine (Tyr) residues. The goal of phosphorylation is to slow down the conformational cycle. Moreover, it also affects the maturation of client proteins as well as the interaction with specific co-chaperones [79,80]. The phosphorylation process is modified by protein phosphatase 5 (PP5 in humans, Ppt1 in yeast cells), which affects the conformation and specificity of HSP90 towards client protein [81]. In the absence of this protein, HSP90 hyperphosphorylation occurs, which leads to the inhibition and impairment of client protein maturation, which was confirmed in the in vivo and in vitro study on yeast cells [82].

The S-nitrosylation process takes place on cysteine (Cys) residues by nitric oxide (NO) within the CTD domain. Its task is to inhibit the activity of HSP90α ATPase and weaken the activation of endothelial nitric oxide synthase (eNOS). Additionally, this type of modification affects the chaperoning activity of HSP90. In turn, ubiquitination inhibits HSP90 functions and causes client protein dissociation [80].

SUMOylation, i.e., the conjugation with SUMO (small ubiquitin-like modifier) HSP90 molecules, takes place on lysine (Lys) residues in the N domain [83]. This process, although little understood, facilitates the recruitment by HSP90 of the AHA1 co-chaperone, which activates ATPase, and facilitates the combination of HSP90 with specific inhibitors, including drugs, which together affects the chaperone cycle [84,85]. Importantly, HSP90 SUMOylation increases in cells undergoing neoplastic transformation, which show increased ATPase activity and a greater affinity for inhibitors, which explains the sensitivity of neoplastic cells to drugs [86].

In addition to the epigenetic modifications, co-chaperones are important regulators of HSP90 function. Their binding sites have been identified in all three domains of HSP90, which account for a large proportion of it. Co-chaperones are proteins that interact with HSP90 and support its function, but their folding process and stability are independent of HSP90. To date, more than 20 different co-chaperones have been identified that are involved in different stages of the HSP90 cycle. Moreover, they induce a different effect of action on HSP90 ATPase and show specificity towards client proteins. Due to some structural similarities, they have been divided into two types: the tetratricopeptide repeat (TPR) domain containing co-chaperones and non-TPR-containing co-chaperones [76,87].

Co-chaperones containing the TPR domain in their structure interact with the MEEVD motif at the C-terminus of the HSP90 through their α-helical domain. One of the best-known co-chaperones is the adapter protein HOP (Sti1 in yeast) (HSP70/HSP90-organizing protein), which acts as a specific linker between the HSP70 and HSP90 protein systems. Its role has also been confirmed as a receptor protein for prion proteins. The main task of HOP is to fold, stabilize and mediate the transfer of client proteins between the two proteins. The transfer of client proteins occurs after the initial recognition and binding of client proteins by HSP70, in cooperation with its J domain containing the HSP40 co-chaperone protein [88,89]. In addition, HOP prevents the closure of the HSP90 conformation, thus keeping it ready to accept and bind effectively to client proteins. This indicates that HOP is a non-competitive inhibitor of ATPase [89,90]. Another co-chaperone protein is protein phosphate 5 (PP5; in yeast Ppt1), which dephosphorylates HSP90 [82,91]. Another group of co-chaperones containing TPR are immunophilins–proteins with the peptidyl-prolyl cis-trans isomerase (PPIase) domain in their structure—cyclophilin (Cyp) (CYP40 in vertebrates, Cpr6 and Cpr7 in yeast)—and FKBP family proteins—FKBP51 and FKBP52 tacrolimus-binding proteins (FK506)–(FK506-binding proteins). These proteins participate in the regulation of the HSP90 conformation cycle, and additionally have a chaperoning activity, and therefore take part in the selection and recruitment of client proteins [76]. Moreover, FKBP51 and FKBP52 participate in the regulation of steroid receptors, affect the transcriptional activity, conformation and transport of proteins. Additionally, they take part in cell differentiation and apoptosis, and are also involved in processes related to tumor progression or telomerase activity [92].

The binding site for co-chaperone proteins, which do not contain TPR, is the NTD and MD domain of HSP90. Among them, the essential CDC37 protein is distinguished, the task of which is to participate in the maturation of kinases. CDC37 is also involved in inhibiting/limiting the closure of the three-dimensional structure of HSP90 and its dimerization. Due to the attachment of HSP90 in the NTD domain, it partially inhibits the activity of ATPase [93]. Another inhibitor of HSP90 activity is the p23 protein (Sba1 in yeast), which stabilizes the closed structure of the HSP90 dimer and thus affects the ATPase activity important for client protein maturation [94,95].

Another protein belonging to this group is the potent activator of HSP90 ATPase activity, AHA1 factor. The stimulation of ATPase activity takes place in a three-step mechanism, through a catalytic loop in the MD domain of HSP90 [96]. The activity of AHA1 can thus modulate the length of the interaction time between HSP90 and the client protein.

In turn, the co-chaperone protein, which does not affect the activity of ATPase, is the small glutamine-rich tetratricopeptide repeat-containing protein alpha (SGTA), which interacts with the NTD HSP90 and HSP70 domain by the folded Chord and Sgt1 (CS) domain. This protein plays a significant role in regulating the activity of the androgen receptor. The interaction of HSP90 and HSP70 with SGTA promotes the reduction of AR signaling through its retention in the cytoplasm and regulation of ligand sensitivity [97]. Reduced SGTA expression is observed during prostate cancer progression, where SGTA is involved in the sensitization of tumor cells to hormonal signaling. An increase in the AR/SGTA ratio in metastatic prostate cancer cells, as compared to primary PCa tumor cells, can cause the reduced control of AR function, and thus exacerbate PCa progression through the receptor [98].

## 5. HSP90 and Prostate Cancer, and Benign Prostatic Hyperplasia

To date, over 200 client proteins have been identified for HSP90. Among them, there are oncoproteins, e.g., the kinases and transcription factors involved in the initiation of the neoplastic process and tumor growth. In neoplastic cells, the client proteins of HSP90 are involved in the transmission of the oncogenic signal, among others, through epithelial growth receptors. In addition, they also participate in the process of angiogenesis (through VEGF (vascular endothelial growth factor) receptors), have an anti-apoptotic effect (PKB, protein kinase B) and can mediate the metastasis process of neoplastic cells (with the participation of MMP2, matrix metallopeptidase 2) [99]. The main role of HSP90 family proteins in the neoplastic process is to control the stabilization of oncogenic client proteins and the regulation of the active state [100,101]. Moreover, depending on HSP90, neoplastic cells maintain their oncogenic activity; additionally, this protein acts as a buffer for cellular stress, increased in the course of the neoplastic process [102]. HSP90 also affects the stabilization of the resistance of cancer cells to hormonal therapy, which was confirmed in the study on human breast cancer models [103]. The increase in HSP90 expression is associated with the progression of neoplastic disease and reduces the chance of survival in breast and lung cancer and in the neoplasms of the gastrointestinal tract [101]. On the other hand, the stimulated inactivation of HSP90 paralogs located in different cell compartments has an antitumor effect and regulates calcium homeostasis [104].

**Figure 4:**
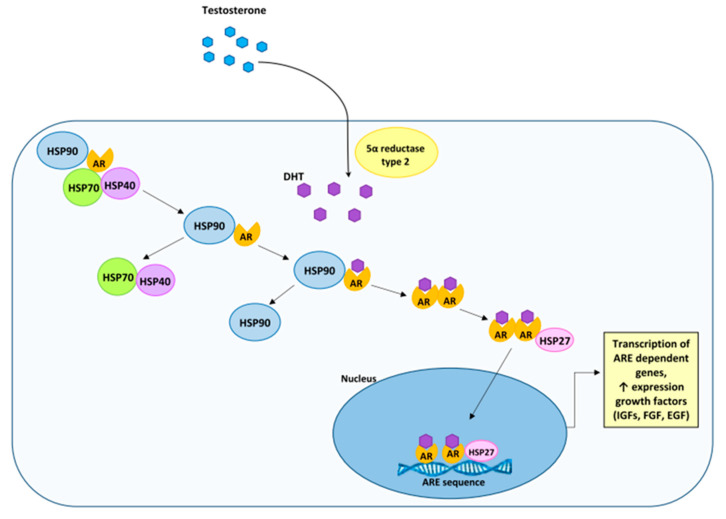
Androgen-AR action in the prostate cells. HSPs regulate AR signaling. A complex of chaperones, including HSP90, associates with the AR to expedite and maintain its high affinity binding conformation, thus allowing for DHT interaction. Testosterone (T) and DHT bind to AR and promote the association of AR coregulators. AR dimer then translocates to the nucleus and binds to AREs sequences in the promoter regions of target genes to induce cell proliferation and apoptosis. IGF, insulin-like growth factor; FGF, fibroblast growth factor and EGF, epidermal growth factor.

The essential function of HSP90 is to protect AR against possible degradation, and thus it contributes to the maintenance of the correct conformation and a high degree of affinity of this receptor for the ligand. AR, which complexes with HSP 90, adopts a conformation that shows a high affinity for the ligand and a low one for DNA. Testosterone or dihydrotestosterone entering the target cell binds with AR, which results in the initiation of a number of intracellular events, such as the detachment of HSP90 from AR, phosphorylation of AR and dimerization of two ARs. The AR homodimer complexes migrate from the cytoplasm to the nucleus where they then look for a specific nucleotide sequence ARE (androgen response element) and bind to DNA. The binding of AR homodimer complexes to the ARE sequence can induce or inhibit the transcription of particular genes (Figure 4).

Clients of HSP90 are proteins, such as protein kinase B (AKT), kinases ERK1 (extracellularly regulated kinases) and ERK2, receptor tyrosine-protein kinase erbB-2 (ERBB2), proto-oncogene tyrosine-protein kinase Src (p60-Src), cyclin-dependent kinases (CDKs) and survivin [20]. In the immunohistochemical study, it was shown that the immunoexpression of HSP90 in the prostate tissue of PCa patients significantly correlates with the stage of prostate cancer, according to the Gleason scale and pTNM classification. In addition, the increase in HSP90 expression was accompanied by an increase in the immunoexpression of IL-10, which is produced by tumor cells in order to induce immunosuppression and avoid immunological surveillance [105].

In the study conducted on cell models (murine and human), it was shown [106] that the use of HSP90 inhibitors (ganetespib and onalespib) in different CRPC genotypes and phenotypes affects the inhibition of oncogenic cell signaling mechanisms regulating tumor growth and development. The use of ganetespib directly reduces the stability of HSP90 client–AKT protein, a key component of the PI3K/AKT/mTOR pathway. Moreover, this inhibitor reduces the level of AR expression. Together, these data indicate that the simultaneous inhibition of the two pathways (AR and PI3K) can positively influence the prevention of the therapeutic resistance of PCa cells [106].

The heat shock protein belonging to the HSP90 family is the mitochondrial tumor necrosis factor receptor-associated protein 1 (TRAP1), which has been characterized as a key metabolic regulator in neoplastic cells. In addition, it is also involved in the process of apoptosis, and, furthermore, it also participates in many signaling pathways in the cell; it acts as a protein that disrupts the cell cycle, increases cell mobility and promotes the metastasis of neoplastic cells. Therefore, TRAP1 turns out to be an important therapeutic target in oncotherapy [107]. In the study by Leav et al. [108], TRAP1 has been shown to be highly expressed in prostate cancer (in human high-grade prostatic intraepithelial neoplasia, Gleason grades 3–5 prostatic adenocarcinomas and metastatic prostate cancer), but undetectable in healthy prostate or benign prostate hyperplasia. The inhibition of TRAP1 induction increases the apoptosis process in prostate cancer cells, which suggests that TRAP1 inhibitors (gamitrinibs (GA), mitochondrial matrix inhibitors) can be used in therapy for patients with advanced prostate glands [108].

Another HSP90 isoform expressed in prostate cancer cells is the protein GRP94 (glucose-regulated protein 94 kDa) found in the ER of cells. This protein is involved in the proper process of protein folding, transport, degradation and in ensuring cell survival during ER stress [109]. Moreover, it is involved in many signaling pathways related to the apoptosis and proliferation process (MAPK and AKT/S6 signaling pathways). In the study by Lu et al. [110], it has been shown that GRP94 along with another chaperone protein, GRP78, are expressed in the cytoplasm and cell membrane in prostate cancer tissue cells, while in benign prostate hyperplasia tissue the expression is negligible. It has been proven that the simultaneous silencing of GRP78 and GRP94 expression with the use of small interfering RNAs (siRNAs) in PCa cells, increases the apoptosis process, by increasing the expression of the Bax protein and significantly inhibiting the migration of tested cancer cells, as a result of a significant inhibition of vimentin expression [110].

In prostate cancer cells, HSP90 positively regulates AR stability and activity, and its inhibition will induce androgen receptor degradation. In CRPC patients, signaling by the AR-FL receptor and its splicing variant AR-V7 plays a significant role in the development of resistance to hormone therapy. Targeting the therapy to AR-FL and AR-V7 can prove to be a strategy to help overcome ADT. In the study by Moon et al. [111], it was found that bruceantin (BCT), a natural substance with antimalarial properties, acts as an inhibitor of the transcriptional activity of AR. The activity of BCT is based on the mechanism of disruption of HSP90 interaction with AR-FL/AR-V7, by the direct binding to HSP90. The result of the formation of such a complex is the inhibition of the chaperone function of HSP90, what leads to degradation of AR-FL/AR-V7 through the ubiquitin–proteasome system [111].

In the study by Ferraldeschi et al. [112], the effect of HSP90 inhibition on prostate cancer cells that show AR-V7 expression was also analyzed. In vitro studies have confirmed that first generation HSP90 inhibitors (tanespimycin and alvespimycin) and second generation ones (onalespib), inhibit tumor cell growth and induce the degradation of client proteins, including AR-FL, AKT and GR (glucocorticoid receptor). It was also found that the inhibition of HSP90 decreased the expression of the AR-V7 variant. However, for AR-V7 to function, unlike AR-FL, the direct interaction with the HSP90 is not required. Nevertheless, it has been observed that the inhibition of HSP90 disrupts pre-mRNA splicing and impairs AR-V7 mRNA formation in ADT resistant cells. This suggests that the inhibition of HSP90 can block the production and upregulation of AR-V7 in CRPC cells [112].

With regard to benign prostatic hyperplasia, there are few studies investigating the effect of HSP90 on the development of BPH. One of the analyses available in the literature is a study carried out on a rat model and in human prostate tissues [113], describing the activity of HSP90 as an autoantigen that binds to the IgG autoantibody and forms an antigen–antibody complex that binds to factor C1q, thus activating the classical complement pathway. The results of the research by Hata et al. [113] indicate the participation of complement system activation in the process of promoting prostate hyperplasia.

Another study on the role of HSP90 in the development of BPH was carried out on a murine model with induced prostate hyperplasia and on human cell lines (LNCaP, BPH-1, WPMY-1), where the role of NAD(P)H-quinone oxidoreductase 1 (NQO1) was analyzed, a FAD-dependent flavoprotein, involved in the defense processes in the cell and preventing the degradation of the p53 protein, in the exacerbation of prostate tissue cell hyperplasia [114]. This study showed that the deficiency of the NQO1 enzyme increases the expression of HSP90, which increases the affinity of AR for testosterone and can be responsible for the enlargement of the prostate gland in NQO1–/– mice.

## 6. The Characteristics of HSP70 and HSP40

### 6.1. HSP70

HSP70s are key components of the cellular network of molecular chaperone proteins. They are involved in the various types of folding of newly synthesized proteins in the cell. In addition, the misfolded and aggregated proteins are refolded. HSP70 is also involved in the membrane translocation of organelle proteins and secretory proteins, and controls the activity of regulatory proteins. Folding is accomplished by the transient association of the HSP-binding domain with short hydrophobic peptide fragments (segments) in an ATP-dependent pathway [115].

The human genome encodes thirteen HSPs belonging to the HSP70 family, which are grouped according to their expression mechanism into inducible or constitutive proteins. The most strongly induced proteins are HSPA1A, HSPA1B and HSPA6, while HSC70 (HSPA8) is distinguished as housekeeping or constitutive proteins. Among the HSP70 family, five proteins are strongly associated with the initiation and progression of the neoplastic process: stress-induced HSP70–HSPA1 and HSPA2, KHSA6 (HSP70B) and constitutively expressed HSC70 (HSPA8), and mortalin (HSPA9) and GRP78 (HSPA5) [4].

**Figure 5:**
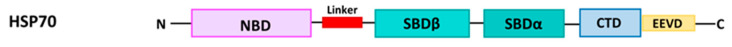
Domain organization of Hsp70. N-terminal, nucleotide-binding domain (NBD), substrate-binding domain (SBDβ), a helical lid domain (SBDα) and a disordered C-terminal domain (CTD) of variable length. In eukaryotic cytosolic and nuclear Hsp70s, the disordered tail frequently ends with a conserved charged motif (Glu-Glu-Val-Asp; EEVD) that interacts with specific cofactors.

The structure and sequence of the homologues of the HSP70 family proteins are highly conserved. There are two main domains in their structure: the 45 kDa N-terminal ATPase domain (nucleotide-binding domain, NBD), which is responsible for the regulation of the activity of these chaperone, and the C-terminal substrate-binding protein domain (SBD) with the size of 25 kDa. The NBD consists of four subdomains: the IA and IB in lobe I and IIA and IIB in lobe II. There is a cleft between the two lobes at the interface of subdomains IIA and IIB, which is the site of ATP binding. The SBD is subdivided into the β-sandwich subdomain (15 kDa) and the C-terminal α-helical subdomain (10 kDa) (Figure 5) [116,117,118]. 

Both domains regulate each other’s activity on the basis of an allosteric effect through the hydrolysis of ATP to ADP. The hydrolysis of ATP of the N-terminal domain increases the substrate binding affinity by the SBD, and thus lowers the substrate exchange rate. On the other hand, the dissociation of ADP generated during ATP hydrolysis and the replacement with new ATP triggers the release of the substrate by the SBD, which in turn increases the rate of substrate exchange. However, the stimulation by the substrates is too low, and the cycle of action of HSP70 is supported by the action of two families of co-chaperones: JDP family proteins (J-domain proteins) and nucleotide exchange factors (NEFs). JDPs (appearing in the literature under the alternative names DnaJ proteins, HSP40 proteins and J-proteins) are a class of heterogeneous multidomain proteins, usually located at the N-terminus. The activity of JDPs is required for the catalysis of ATP hydrolysis. On the other hand, NEFs are involved in the replacement of ADP with ATP, which significantly accelerates the dissociation of ADP [116,119].

### 6.2. HSP40

HSP40, also known in the literature as DnaJ or the J-domain protein, is a chaperone that cooperates with HSP70 in many biological processes; among others, this complex is involved in the synthesis of proteins, their translocation across the cell membrane and the folding process. These proteins were identified by the presence of the highly conserved 70-amino acid, J domain that stimulates the ATPase activity of HSP70. HSP40s exhibit anti-aggregation chaperone activity. It is hypothesized that HSP40 functions as a factor of substrate scanning for HSP70 and a carrier of peptide substrates, due to different substrate preferences [117].

**Figure 6:**
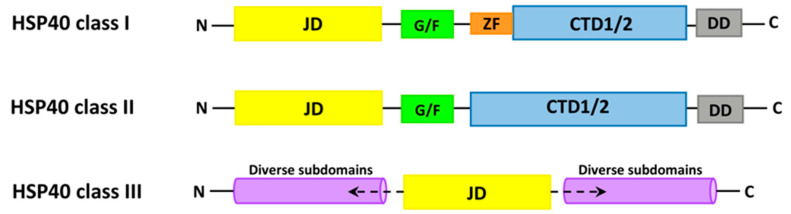
Domain structure of the three HSP40 (DnaJ). Subfamilies: class I, class II and class III. The different domains are marked in the following way: JD, J domain; G/F, Gly-Phe rich region; ZF, Zinc finger (Zn^2+^-binding domain); CTD, C-terminal domain and DD, dimerization domain.

Among HSP40s, three classes have been distinguished: class I (in the literature described as class A), class II (class B) and class III (class C). So far, proteins isolated from different species, the thermus thermophilus type B Hsp40 (ttHsp40), the *E. coli* type B Hsp40 (CbpA), the yeast type A (Ydj1) and type B Hsp40s (Sis1), and the human type B Hsp40 (DNAJB1), have been studied [120]. In contrast, 49 proteins from the DNAJ family have been identified in humans, which have been divided into 3 subclasses: type I (DNAJA, containing 4 proteins), type II (DNAJB, 13 proteins) and type III (DNAJC, 32 proteins) [121]. Each class is made up of characteristic domains. The first class consists of 5 domains: the J domain at the N-terminus, glycine/phenylalanine rich regions (G/F), first carboxy-terminal (CTD1) and second carboxy-terminal (CTD2) with a zinc finger (ZFLR), and the dimerization domain (D). The difference between class I and II is the lack of a zinc finger in the first carboxy-terminal domain. Both carboxy-terminal domains in both classes are necessary for the proper transfer of the peptide substrate. Class III HSP40s only share the J-domain with other classes (Figure 6).

The J-domain found in HSP40s is necessary for the interaction of this protein with HSP70; it is a 70-amino acid sequence composed of 4 helices and a loop region containing the tripeptide histidine, proline and ascorbic acid referred to as the HPD motif. In the experiments in a rat model, the N-terminal HSP70 terminus has been shown to contain a DnaJ-linking portion that connects via the HPD motif to HSP70 [122].

Due to this complex, it is possible to regulate the ATPase activity of HSP70. Once ATP is bound to the N-terminal domain, this domain is in the open conformation with the low affinity for non-native polypeptides. When ATP-to-ADP hydrolysis occurs, the N-terminal domain changes into a closed conformation with a high affinity for non-native polypeptides [123]. Different classes of HSP40 can affect HSP70s by changing their activity depending on the class attached. Class I HSP40 enables the proper folding of HSP70. Class III accompanies HSP70 in proteostasis. HSP40s are essential for the differentiation of HSP70 functions [117]. HSP40 accompanies HSP70 in folding mainly in two planes: by stimulating ATP hydrolysis and providing substrates for HSP70.

HSP40 and HSP70 form a complex in which HSP40 acts as a cochaperone. In this complex, HSP40 regulates HSP70 by inducing its ATPase activity, resulting in the stabilization of the HSP70–peptide complex [124]. More HSP40 isoforms were found to be present in cells than HSP70 ones. At the same time, a lower concentration of DnaJ than HSP70 is required to adequately initiate the folding process of the HSP70 terminus structure. The role of HSP40 in tumor growth, however, is not clearly defined, so the most important is the effect of HSP40 on the activity of HSP70 and the development of its modulators targeting specific locations in cancer research [125]. The HSP40 subclass can function independently of HSP70 to prevent protein aggregation. Studies have shown that upregulation of various HSP40 subclasses is associated with high-grade intraepithelial neoplasia and the maintenance of client proteins stability [124].

The mechanism of interaction between HSP40 and HSP70 is also involved in modulating the activity of AR. First, HSP40 binds to AR, and then recruits HSP70 via the J-domain. Subsequently, in cells, the binding of the J domain affects the ATP cycle through HSP70, which then leads to the formation of a client–chaperone complex with a very high affinity [126]. HSP40 supports HSP70 in locating AR in the cell and accelerates its catalytic cycle [127].

## 7. HSP70 and HSP40 and Prostate Cancer

The chaperone protein belonging to the HSP70 family is GRP78 (also known as HSPA5 or the binding immunoglobulin protein (BiP)), which is constitutively expressed under stressful conditions in the tumor environment. The results of the study by Pootrakul et al. [128] on human prostate cancer tissue and cell lines, showed that GRP78 expression is significantly elevated in metastatic castration-resistant prostate cancer compared to local PCa. Moreover, GPR78 expression was increased in LNCaP-derived cell line C42B castration-resistant cells and in LNCaP cells growing under androgen deficiency conditions, compared to the same cells grown under androgen-rich conditions. This indicates that the overexpression of GPR78 renders the tumor cells resistant to apoptosis and hormone therapy, thereby leading to the growth of a castration-resistant tumor. Moreover, the results of this study also suggest that the increased expression of GPR78 is a predictive factor for prostate cancer recurrence in patients who were diagnosed with the cancer at an early age [128]. The role of GRP78 in the functioning of prostate cancer cells was also confirmed in the study by Cultara et al. [129]. The silencing of GPR78 expression via siRNA in prostate cancer cells (in bone metastatic PCa cell line, PC3) reduces the level of the adhesion protein N-cadherin (N-cad), the participation of which has already been confirmed in metastasis and castration resistance PCa cells. N-cad suppression by GPR78 has been shown to significantly reduce the adherence of prostate cancer cells to osteoblasts. These data suggest that the inhibition of GRP78 can be an appropriate strategy for the treatment of PCa cancer undergoing metastasis into the bone microenvironment. Moreover, strong GRP78 silencing in the cell did not increase cytotoxicity [129].

The mechanism of the effect of HSP70 on prostate cancer cells is based on the direct interaction of the substrate-binding domain of this protein with the NTD domain of AR, as confirmed by the study by Dong et al. [130]. The binding of HSP70 to AR, regulates the expression of the endogenous receptor in prostate cells, and the inhibition of HSP reduces the expression and reduces the transcriptional activity of AR. This study indicates that HSP70 and the use of its inhibitors can be a new therapeutic target for PCa. The currently used drug, enzulamide, inhibits the action of AR by interacting with the LBD domain of the receptor, which is a therapy failure, with cancer cells expressing AR variants that do not contain this domain in their structure. Defining a new way and mechanisms of reducing the activity of AR and its variants is crucial when cells are resistant to the available treatment [130].

HSP70 is also considered as a therapeutic target for CRPC, where anti-tumor activity is achieved through the use of inhibitors of this protein. In the study by Kit et al. [131] conducted on LNCaP95 cells expressing the AR-V7 receptor, it was analyzed how HSP70 inhibitors (quercetin and VER155008) affect the tested cells. The results of this study clearly show that the inhibition of HSP70 reduces the phosphorylation of YB-1 regulating the transcription of both AR-FL and AR-V7, which leads to a reduction in the expression level of the AR variants and an increase in the effectiveness of therapy in CRPC. Moreover, these inhibitors also reduce cell proliferation and increase the percentage of apoptotic cells.

In the study by Moses et al. [132], HSP40s and HSP70s have been shown to be chaperones for AR variants lacking the LBD domain, thereby ensuring their stability and function, which is disadvantageous in the treatment of CRPC patients. Moreover, the chaperone HSP40/HSP70 axis is responsible for the regulation of the GR receptor, the significantly increased expression of which has been confirmed in enzulamide-resistant CRPC, which is an alternative mechanism of tumor cell resistance to AR-targeted therapies. This study confirms that an effective therapy against prostate cancer should be aimed at the inhibition of HSP40s and HSP70s. In this way, the transcriptional activity of FL-AR, ARv7 and GR, the increased expression of which contributes to the development of resistance and therapy failure in CRPC, would be limited [132].

Another study [127], conducted in a cellular model, describes the mechanisms by which the chaperone proteins, HSP40 and HSP70, regulate the aggregation, activation and control of AR. These protein chaperones hold the AR in an inactive conformation. In the presence of androgens, they are released, which allows the receptor to activate and become susceptible to aggregation. HSP40s and HSP70s recognize the NTD domain region of the AR receptor, including the FQNLF motif, which upon activation interacts with the AR ligand-binding domain (LBD). This indicates that the regulation of AR activation is mediated by the competition between chaperone proteins and LBD for binding to the FQNLF motif. These data indicate that targeting the therapy to HSP70, and at the same time to HSP40, will promote the clearance of misfolded proteins, and not in a manner that is similar to the case of HSP90–client proteins [127].

## 8. Conclusions

The results of many studies suggest that there are a number of dynamic interactions between client proteins and the HSP40/HSP70/HSP90 chaperone machinery in prostate cancer cells, including CRPC, which is consistent with the pleiotropic nature of molecular chaperones. Importantly, these data also support the need for further research to target the HSP40/HSP70 chaperoning axis as an alternative multivariate therapeutic strategy for the treatment of CRPC. Understanding the mechanisms of the regulation of genes encoding steroid receptors, including AR and GR, with the participation of chaperone proteins, allows for a better understanding of their role in receptor-dependent diseases. This is especially important in the context of prostate diseases, mainly prostate cancer, in which AR is an important therapeutic target because the expression of genes related to tumor cell proliferation and cell proliferation in BPH are controlled through this receptor. Therefore, recently, more and more research has focused on the heat shock protein inhibitors HSP90, HSP70 and HSP40, which can inhibit both the full-length AR and its splicing variants as potential cancer therapy targets in enzalutamide-resistant prostate cancer, which is now a serious clinical challenge.

## Data Availability

Not applicable.
